# Supplementary material for: Spatiotemporal proteomic profiling of cellular responses to NLRP3 agonists
Source: bioRxiv. 2024 Apr 20:2024.04.19.590338. Preprint. [Version 1] doi: 10.1101/2024.04.19.590338 (PMC11042255; doi:10.1101/2024.04.19.590338)
Supplement: 1 — Supplemental tables are hosted on Zenodo: 10.5281/zenodo.10975767 Table S1. Whole cell proteomics data and analysis (associated with Figure 2). Table S2. LysoIP proteomics data and analysis (associated with Figure 3, Figure 3—Supporting Data Figure 1). Table S3. MitoIP proteomics data and analysis (associated with Figure 3, Figure 3—Supporting Data Figure 3). Table S4. EndoIP proteomics data and analysis (associated with Figure 4, Figure 4—Supporting Data Figure 1). Table S5. GolgiIP (nigericin, CL097) proteomics data and analysis (associated with Figure 5, Figure 5—Supporting Data Figure 1). Table S6. GolgiIP (monensin, retro-2) proteomics data and analysis (associated with Figure 5, Figure 5—Supporting Data Figure 4). Table S7. APEX2 timecourse (P4C, nigericin) proteomics data and analysis (associated with Figure 6, Figure 6—Supporting Data Figure 3). Table S8. APEX2 timecourse (P4C, CL097) proteomics data and analysis (associated with Figure 6, Figure 6—Supporting Data Figure 4). Table S9. APEX2 timecourse (Nlrp3, nigericin) proteomics data and analysis (associated with Figure 6, Figure 6—Supporting Data Figure 5). Table S10. APEX2 timecourse (Nlrp3, CL097) proteomics data and analysis (associated with Figure 6, Figure 6—Supporting Data Figure 6). Table S11. Meta cluster data across timecourse data, and linear model timecourse data (associated with Figure 6). Table S12. APEX2 combined (P4C and Nlrp3; CL097 and nigericin) proteomics data and analysis (associated with Figure 7). Table S13. APEX2 priming (Nlrp3 and P4C, ±LPS) proteomics data and analysis (associated with Negative Data Figure 5). Table S14. Organellar annotations (associated with multiple figures). Table S15. MS data acquisition parameters and key resources used or made in this study. Includes cell lines, plasmids, sgRNA sequences, antibodies, and other reagents. [file NIHPP2024.04.19.590338V1-supplement-1.pdf]

bioRxiv preprint doi: https://doi.org/10.1101/2024.04.19.590338; this version posted April 20, 2024. The copyright holder for this preprint (which was not certified by peer review) is the author/funder, who has granted bioRxiv a license to display the preprint in perpetuity. It is made available under aCC-BY 4.0 International license.

Supporting figures

Figure 2 supporting figure

bioRxiv preprint doi: https://doi.org/10.1101/2024.04.19.590338; this version posted April 20, 2024. The copyright holder for this preprint (which was not certified by peer review) is the author/funder, who has granted bioRxiv a license to display the preprint in perpetuity. It is made available under aCC-BY 4.0 International license.

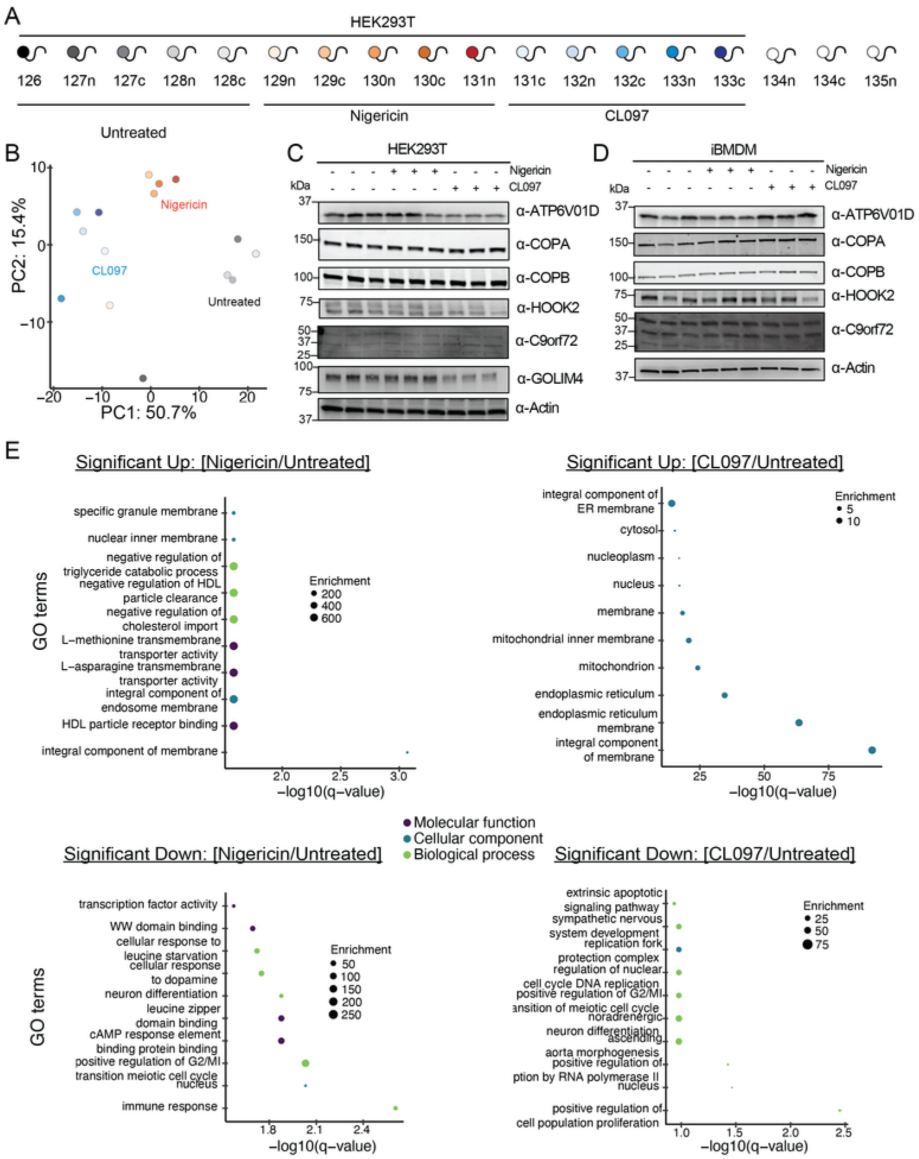

Figure 2—Supporting Figure 1. TMT design, GO term analysis, and other supporting data for the whole cell proteomics experiment.
(A) TMTplex experimental design. Cells were treated with the indicated compounds (nigericin: 20 μM, 30 min; CL097: 75 μg/mL, 1 h) prior to harvesting lysates for whole cell proteomics. n=5 biological replicates per condition.
(B) Principal component analysis (PCA) of the data colored as in (A).
(C-D) Western blots from (C) HEK293T cells and (D) iBMDMs treated with nigericin (20 μM, 30 min) or CL097 (75 μg/mL, 1 h). n=3 biological replicates shown.
(E) GO-term analysis of proteins significantly changing (q > 0.05, Log_2FC > |0.5|) in response to the indicated treatment.
To Figure 2: https://harperlab.pubpub.org/pub/nlrp3#n7w50fsfetx

Figure 3 supporting figures

bioRxiv preprint doi: https://doi.org/10.1101/2024.04.19.590338; this version posted April 20, 2024. The copyright holder for this preprint (which was not certified by peer review) is the author/funder, who has granted bioRxiv a license to display the preprint in perpetuity. It is made available under aCC-BY 4.0 International license.

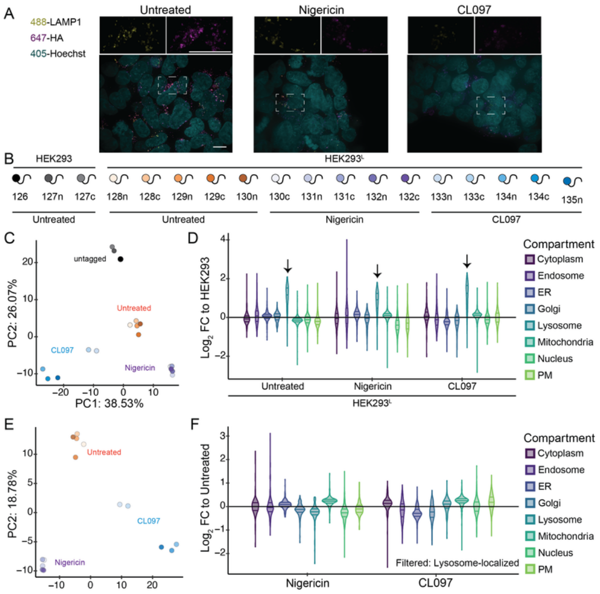

Figure 3—Supporting Figure 1. Experimental validation and design for the LysoIP experiment.

(A) The LysoTag (TMEM192-HA) colocalizes with the lysosomal marker LAMP1 and maintains colocalization following treatment. HEK293^{L} cells were treated with nigericin (20 µM, 30 min) or CL097 (75 µg/mL, 1 h), fixed, and immunostained with the indicated antibody and Hoechst. Maximum intensity projection images (z=8 µM, 29 steps), representative of n > 6 fields of view. Scale bar (left panel), 10 µm.

(B) TMTplex experimental design. Cells were treated with the indicated compounds (nigericin, 20 µM, 30 min; CL097, 75 µg/mL, 1 h) prior to LysoIP on anti-HA magnetic beads. The parental untagged HEK293 cell line serves as a background control, whereas the endogenously tagged HEK293 cell line (HEK293^{L}) facilitates LysoIP.

(C) Principal component analysis (PCA) colored as in (B) with all channels included. Replicates of a given condition correlate well.

(D) Violin plots depicting the $\log_2$ fold change (FC) values for the indicated subcellular compartment in aggregate for the indicated LysoIP (HEK293^{L}) compared to the background control (HEK293). Lysosomal proteins are enriched over all organellar groups for each condition, validating the approach.

(E) PCA as colored as in (B) with background samples excluded. Replicates for a given condition cluster well.

(F) Violin plots depicting the $\log_2$ FC values for the indicated subcellular compartment and given treatment condition (nigericin or CL097) compared to untreated (LysoTag) cells. Proteins were first filtered for significant lysosomal localization (any 293L/293: $q < 0.05$, Log_2 FC > 0.5)

To Figure 3: https://harperlab.pubpub.org/pub/nlrp3#nyjwc0zexqn

bioRxiv preprint doi: https://doi.org/10.1101/2024.04.19.590338; this version posted April 20, 2024. The copyright holder for this preprint (which was not certified by peer review) is the author/funder, who has granted bioRxiv a license to display the preprint in perpetuity. It is made available under aCC-BY 4.0 International license.

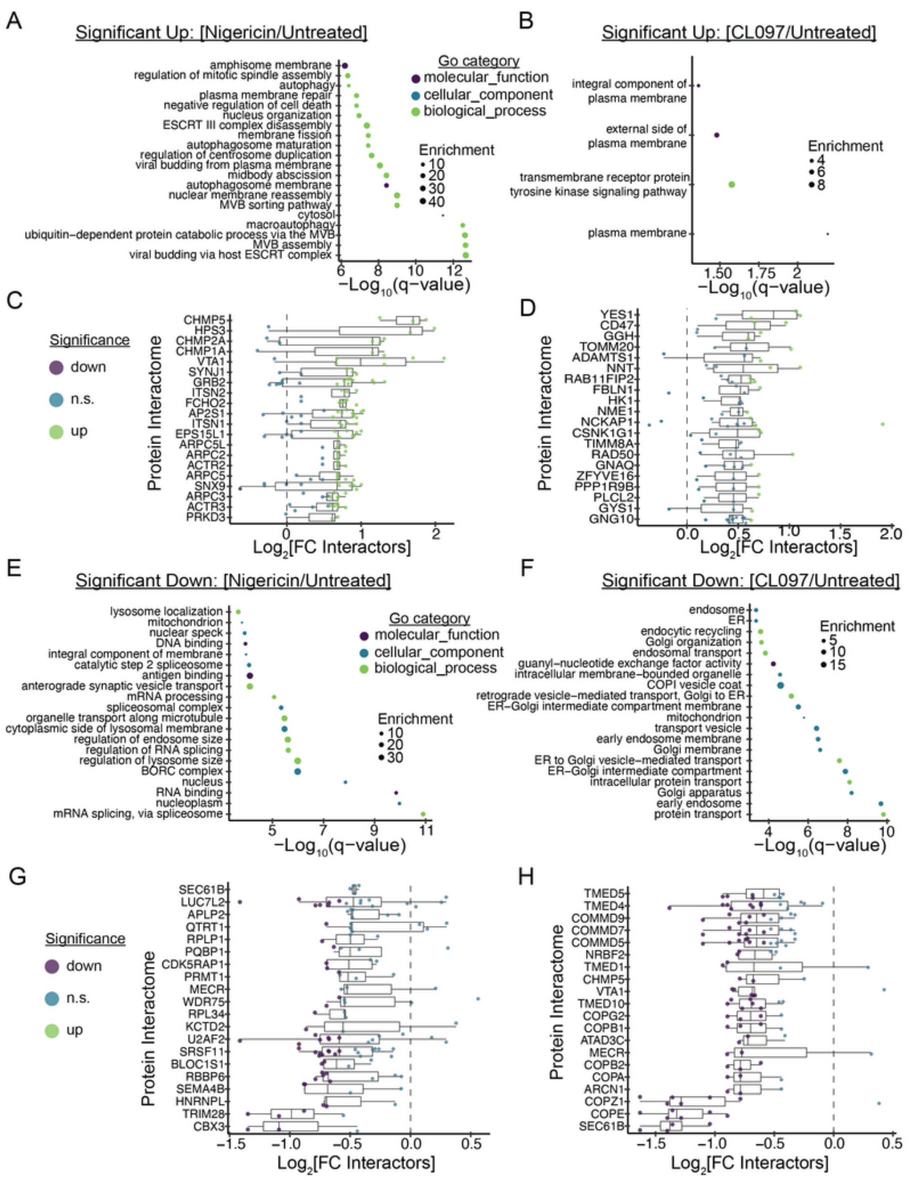

Figure 3—Supporting Figure 2. Protein group analysis for the LysoIP experiment.

(A-B) GO-term analysis for proteins that significantly increased in the indicated LysoIP versus untreated cells (q < 0.05, $Log_2FC > 0.5$).

(C-D) Analysis of the top 20 Bioplex protein interactomes (>3 proteins detected) positively enriched in the indicated experiment.

(E-F) GO-term analysis for proteins that significantly decreased in the indicated LysoIP versus untreated cells (q > 0.05, Log_2 FC < -0.5).

(G-H) Analysis of the top 20 Bioplex protein interactomes (>3 proteins detected) depleted in the indicated experiment.

To Figure 3: https://harperlab.pubpub.org/pub/nlrp3#nyjwc0zexqn

bioRxiv preprint doi: https://doi.org/10.1101/2024.04.19.590338; this version posted April 20, 2024. The copyright holder for this preprint (which was not certified by peer review) is the author/funder, who has granted bioRxiv a license to display the preprint in perpetuity. It is made available under aCC-BY 4.0 International license.

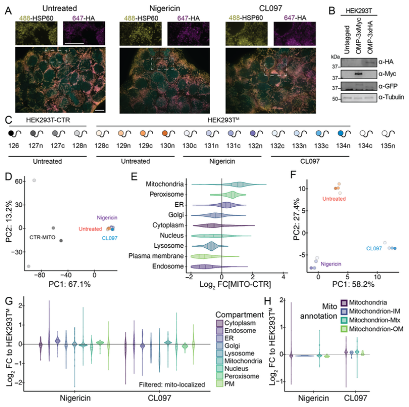

Figure 3—Supporting Figure 3. Experimental validation and design for the MitoIP experiment.

(A) The MitoTag (OMP25-eGFP-3xHA) colocalizes with the mitochondrial marker HSP60 and maintains colocalization following treatment. HEK293T^{M} cells were treated with nigericin (20 µM, 30 min) or CL097 (75 µg/mL, 1 h), fixed, and immunostained with the indicated antibody and Hoechst. Maximum intensity projection images (z=8 µM, 29 steps), representative of n > 6 fields of view. Scale bar (left panel), 10 µm.

(B) TMTplex experimental design. Cells were treated with the indicated compounds (nigericin, 20 µM, 30 min; CL097, 75µg/mL, 1 h) prior to MitoIP on anti-HA magnetic beads. 3xMyc-tagged HEK293T cells serve as the background control (HEK293T-CTR), whereas the 3xHA-tagged HEK293T cell line (HEK293T^{M}) facilitates MitoIP.

(C) Western blots of the HEK293T-CTR (myc- and eGFP-tagged) and HEK293T^{M} (HA- and eGFP-tagged) cell lines confirming protein expression at similar levels.

(D) Principal component analysis (PCA) colored as in (C) with all channels included. Background control IPs separate well from MitoIPs.

(E) Violin plots depicting the $\log_2$ fold change (FC) values for the indicated subcellular compartment in aggregate for the untreated MitoIP (HEK293T^{M}) compared to the background control (HEK293T-CTR). Mitochondrial proteins are enriched over all organellar groups for each condition, validating the approach.

(F) PCA colored as in (C) with background samples excluded. Replicates of a given condition cluster well.

(G) Violin plots depicting the $\log_2$FC values for the indicated subcellular compartment and the given treatment condition (nigericin or CL097) compared to untreated (MitoTag) cells. Proteins were first filtered for significant mitochondrial localization (any 293T^{M}/293T^{Control}: q < 0.05, $\log_2$ FC > 0.5).

(H) Violin plots depicting the $\log_2$FC values for the indicated mitochondrial annotation and the given treatment condition compared to untreated (MitoTag) cells. Proteins were first filtered for significant mitochondrial localization (any 293T^{M}/293T^{Control}: q < 0.05, $\log_2$FC > 0.5).

To Figure 3: https://harperlab.pubpub.org/pub/nlrp3#nyjwc0zexqn

bioRxiv preprint doi: https://doi.org/10.1101/2024.04.19.590338; this version posted April 20, 2024. The copyright holder for this preprint (which was not certified by peer review) is the author/funder, who has granted bioRxiv a license to display the preprint in perpetuity. It is made available under aCC-BY 4.0 International license.

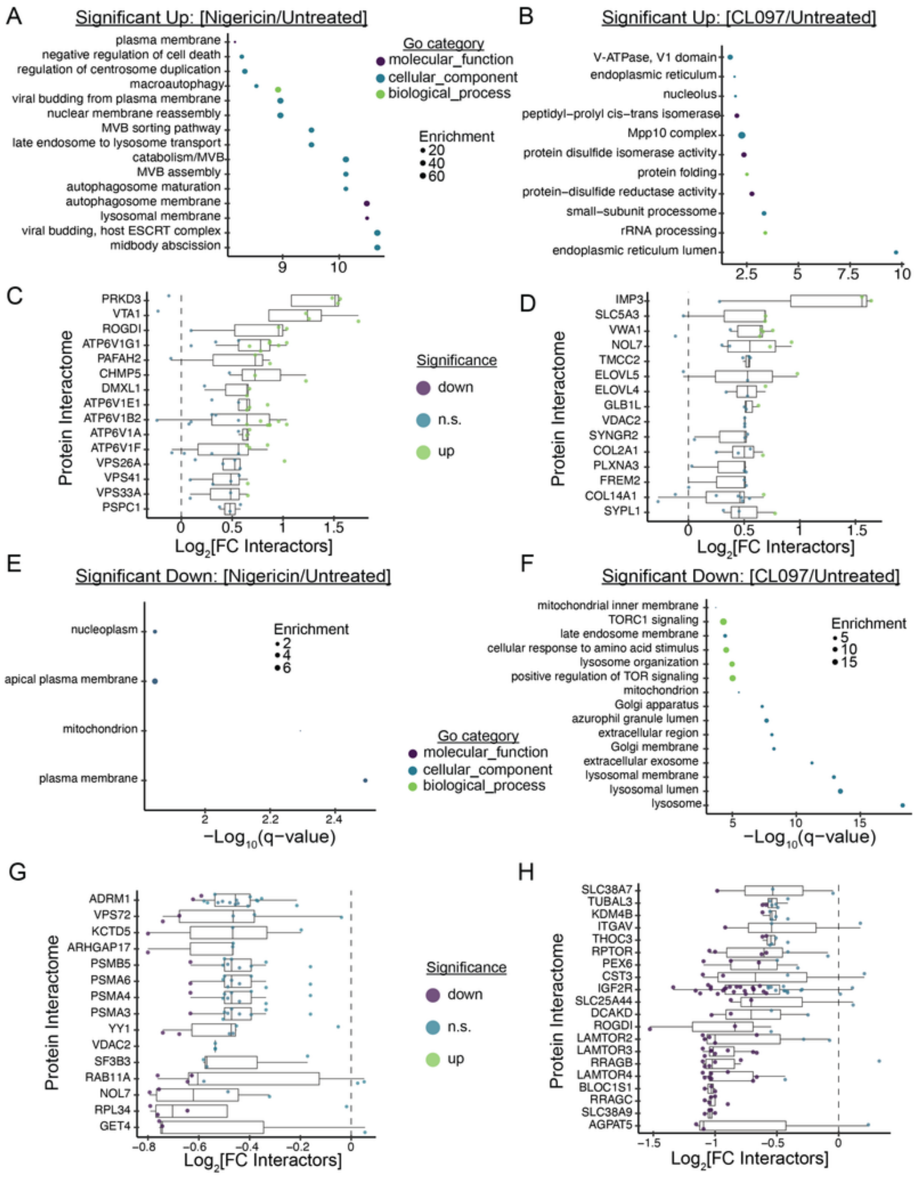

Figure 3—Supporting Figure 4. Protein group analysis for the MitoIP experiment.
(A-B) GO-term analysis for proteins that significantly increased in the indicated MitoIP versus untreated cells ($q < 0.05$, Log_2 FC > 0.5).

(C-D) Analysis of the top 20 Bioplex protein interactomes (>3 proteins) positively enriched in the indicated experiment.

(E-F) GO-term analysis for proteins that significantly decreased in the indicated MitoIP versus untreated cells ($q > 0.05$, Log_2 FC < -0.5).

(G-H) Analysis of the top 20 Bioplex protein interactomes (>3 proteins) depleted in the indicated experiment.

To Figure 3: https://harperlab.pubpub.org/pub/nlrp3#nyjwc0zexqn

Figure 4 supporting figures

bioRxiv preprint doi: https://doi.org/10.1101/2024.04.19.590338; this version posted April 20, 2024. The copyright holder for this preprint (which was not certified by peer review) is the author/funder, who has granted bioRxiv a license to display the preprint in perpetuity. It is made available under aCC-BY 4.0 International license.

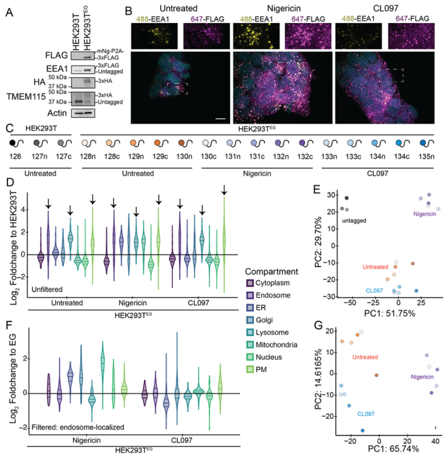

Figure 4—Supporting Figure 1. Experimental validation and design for the EndoIP experiment.

(A) Western blot validation of HEK293T^{EG} cells. HEK293T cells were endogenously tagged with the EndoTag (3xFLAG-EEA1) and the GolgiTag (TMEM115-3xHA), then immunobloted with the indicated antibodies.

(B) The EndoTag (3xFLAG-EEA1) localizes to endosomes and maintains localization following treatment. HEK293^{EG} cells were treated with nigericin (20 µM, 30 min) or CL097 (75 µg/mL, 1 h), fixed, and immunostained with the indicated antibody and Hoechst. Maximum intensity projection images (z=8 µM, 29 steps), representative of n > 6 fields of view. Scale bar (left panel), 10 µm.

(C) TMTplex experimental design. Cells were treated with the indicated compounds (nigericin, 20 µM, 30 min; CL097, 75 µg/mL, 1 h) prior to EndoIP on anti-FLAG magnetic beads. The parental untagged HEK293T cell line serves as a background control, whereas the endogenously tagged HEK293T cell line (HEK293T^{EG}) facilitates EndoIP.

(D) Violin plots depicting the $\log_2$FC values for the indicated subcellular compartment in aggregate for the indicated EndoIP compared to the background control (HEK293T). Endosomal, lysosomal, and plasma membrane proteins (groups that likely traffic through endosomes) are enriched over all organellar annotations for each condition (indicated by arrows), validating the approach.

(E) Principal component analysis (PCA) colored as in (C) with all channels included. Replicates of a given condition correlate well.

(F) Violin plots depicting the $\log_2$ FC values for the indicated organelle annotation and the given treatment condition compared to untreated (EndoTag) cells. Proteins were first filtered for significant endosomal localization (any 293T^{EG}/293T: $q < 0.05$, Log_2 FC > 0.5).

(G) PCA colored as in (C) with background samples excluded. Proteins were first filtered for significant endosomal localization (any 293T^{EG}/293T: $q < 0.05$, Log_2 FC > 0.5). Replicates of a given condition correlate well.

To Figure 4: https://harperlab.pubpub.org/pub/nlrp3#n75ph0ar597

bioRxiv preprint doi: https://doi.org/10.1101/2024.04.19.590338; this version posted April 20, 2024. The copyright holder for this preprint (which was not certified by peer review) is the author/funder, who has granted bioRxiv a license to display the preprint in perpetuity. It is made available under aCC-BY 4.0 International license.

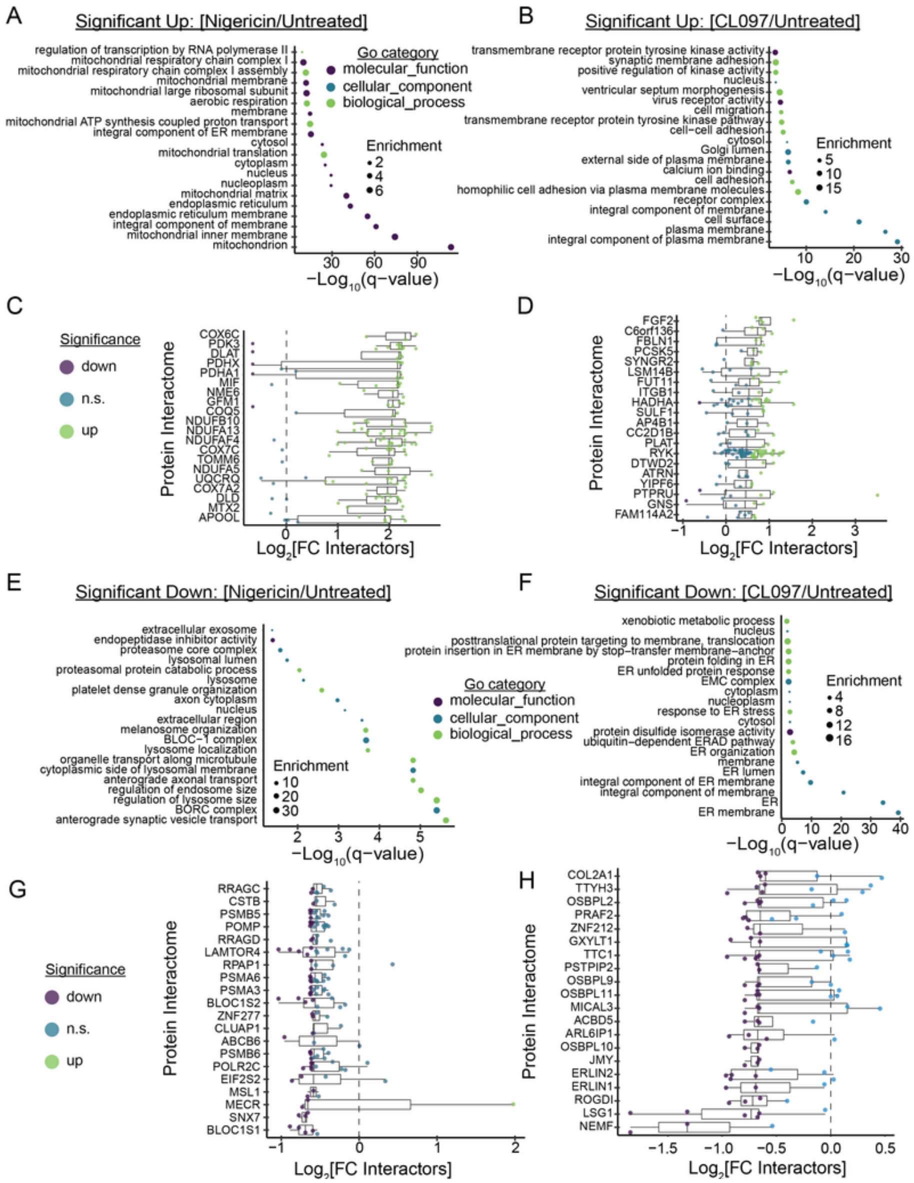

Figure 4—Supporting Figure 2. Protein group analysis for the EndoIP experiment. (A-B) GO-term analysis for proteins that significantly increased in the indicated EndoIP versus untreated cells ($q < 0.05$, Log_2 FC > 0.5).

(C-D) Analysis of the top 20 Bioplex protein interactomes (>3 proteins) positively enriched in the indicated experiment.

(E-F) GO-term analysis for proteins that significantly decreased in the indicated EndoIP versus untreated cells ($q < 0.05$, Log_2 FC < -0.5).

(G-H) Analysis of the top 20 Bioplex protein interactomes (>3 proteins) depleted in the indicated experiment.

To Figure 4: https://harperlab.pubpub.org/pub/nlrp3#n75ph0ar597

Figure 5 supporting figures

bioRxiv preprint doi: https://doi.org/10.1101/2024.04.19.590338; this version posted April 20, 2024. The copyright holder for this preprint (which was not certified by peer review) is the author/funder, who has granted bioRxiv a license to display the preprint in perpetuity. It is made available under aCC-BY 4.0 International license.

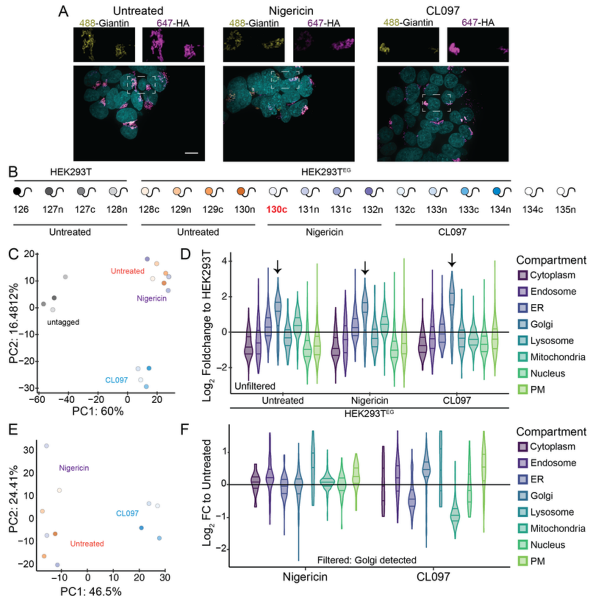

Figure 5—Supporting Figure 1. Experimental validation and design for the GolgiIP (nigericin, CL097) experiment.

(A) The GolgiTag (TMEM115-3xHA) localizes to Golgi and maintains localization following treatment. HEK293T^{EG} cells were treated with nigericin (20 μM, 30 min) or CL097 (75 μg/mL, 1 h), fixed, and immunostained with the indicated antibody and Hoechst. Maximum intensity projection images (z=8 μM, 29 steps), representative of n>4 fields of view. Scale bar (left panel), 10 μm.

(B) TMTplex experimental design. Cells were treated with the indicated compounds (nigericin, 20 μM, 30 min; CL097, 75μg/mL, 1 h) prior to GolgiIP on anti-HA magnetic beads. The parental untagged HEK293T cell line serves as a background control, whereas the endogenously tagged HEK293T cell line (HEK293T^{EG}) facilitates GolgiIP. Channel 130n (bold red) is an outlier (insufficient material following IP as detected by the ratio check) and was excluded from analysis.

(C) Principal component analysis (PCA) colored as in (B) with all channels included. Replicates of a given condition correlate well.

(D) Violin plots depicting the $\log_2$ fold change (FC) values for the indicated subcellular compartment in aggregate for the indicated GolgiIP compared to the background control (HEK293T). GolgiIP predominantly enriches for Golgi proteins (indicated by arrows) across all experimental conditions.

(E) PCA colored as in (B) with background samples excluded. Replicates of a given condition correlate well.

(F) Violin plots depicting the log2 FC values for the indicated organelle annotation and the given treatment condition compared to untreated (GolgiTag) cells. Proteins were first filtered for significant Golgi localization (any 293T^{EG}/293T: $q < 0.05$, $\text{Log}_2\text{FC} > 0.5$).

To Figure 5: https://harperlab.pubpub.org/pub/nlrp3#nkrmiksruzv

bioRxiv preprint doi: https://doi.org/10.1101/2024.04.19.590338; this version posted April 20, 2024. The copyright holder for this preprint (which was not certified by peer review) is the author/funder, who has granted bioRxiv a license to display the preprint in perpetuity. It is made available under aCC-BY 4.0 International license.

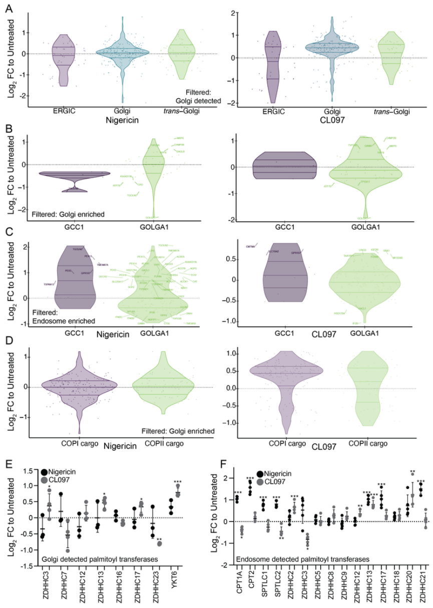

Figure 5—Supporting Figure 2. Golgi-related organellar terms and palmitoyltransferases. (A) Violin plots depicting the log_2 fold change (FC) values for the indicated Golgi/ER subcompartment in aggregate for the indicated GolgiIP compared to the untreated control IP (HEK293T^{EG}). Only Golgi enriched proteins are displayed ($Log_2FC > 0.5$, $q < 0.05$ compared to the untagged control). Only Golgi enriched proteins are displayed (any 293T^{EG}/293T: $q < 0.05$, $Log_2FC > 0.5$). Annotations reported in Hein*, Peng*, Todorova*, McCarthy*, Kim*, and Liu* *et al.* (Leiden cluster annotations) [84].

(B-C) Violin plots depicting the log_2FC values for GOLGA1/GCC1 cargo proteins in aggregate for the indicated (B) GolgiIP or (C) EndoIP compared to the untreated control IP (HEK293T^{EG}). Only proteins enriched over the matched negative control IP are displayed (any 293T^{EG}/293T: $q < 0.05$, $Log_2FC > 0.5$). Significantly changing proteins are indicated. Annotations reported in Shin *et al.* [132].

(D) Violin plots depicting the log_2FC values for COPI/COPII cargo proteins in aggregate for the indicated GolgiIP compared to the untreated positive control IP (HEK293T^{EG}). Annotations reported in Adolf* and Rhiel* *et al.* [197].

(E-F) Log_2FC values for (E) Golgi-enriched or (F) endosome-enriched ($Log_2FC > 0.5$, $q < 0.05$ compared to the untagged control) palmitoyl transferases in response nigericin (20 μM, 30 min) or CL097 (75 μg/mL, 1 h) compared to the untreated positive control. Error bars represent the standard deviation from n=3, 4, or 5 biological replicates. *P* values (B,C,E,F) were calculated from the Student's t-test (two sided) and adjusted for multiple hypothesis correction using the Benjamini–Hochberg approach (q-value) with MSstats. *q<0.05, **q<0.01, ***q<0.001.

To Figure 5: https://harperlab.pubpub.org/pub/nlrp3#nkrmiksruzv

bioRxiv preprint doi: https://doi.org/10.1101/2024.04.19.590338; this version posted April 20, 2024. The copyright holder for this preprint (which was not certified by peer review) is the author/funder, who has granted bioRxiv a license to display the preprint in perpetuity. It is made available under aCC-BY 4.0 International license.

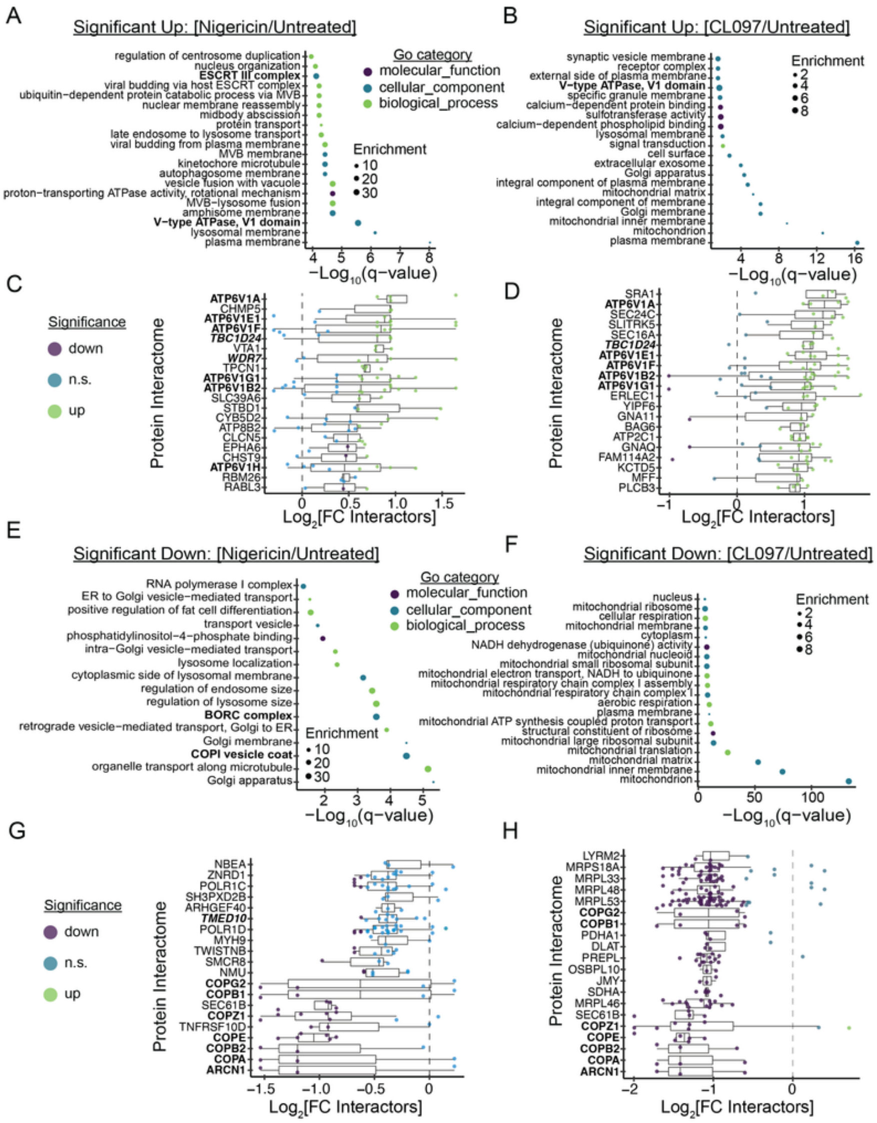

Figure 5—Supporting Figure 3. Protein group analysis for the GolgiIP experiment. (A-B) GO-term analysis for proteins that significantly increased in the indicated GolgiIP versus untreated cells ($Log_2FC > 0.5$, $q < 0.05$).

(C-D) Analysis of the top 20 Bioplex protein interactomes (>3 proteins) positively enriched in the indicated experiment. V-ATPase interactors are in bold and italicized.

(E-F) GO-term analysis for proteins that significantly decreased in the indicated LysoIP versus untreated cells ($Log_2FC < -0.5$, $q < 0.05$).

(G-H) Analysis of the top 20 Bioplex protein interactomes (>3 proteins) depleted in the indicated experiment. COPI subunits are in bold, and their COPI-associated proteins are in bold and italicized.

To Figure 5: https://harperlab.pubpub.org/pub/nlrp3#nkrmiksruzv

bioRxiv preprint doi: https://doi.org/10.1101/2024.04.19.590338; this version posted April 20, 2024. The copyright holder for this preprint (which was not certified by peer review) is the author/funder, who has granted bioRxiv a license to display the preprint in perpetuity. It is made available under aCC-BY 4.0 International license.

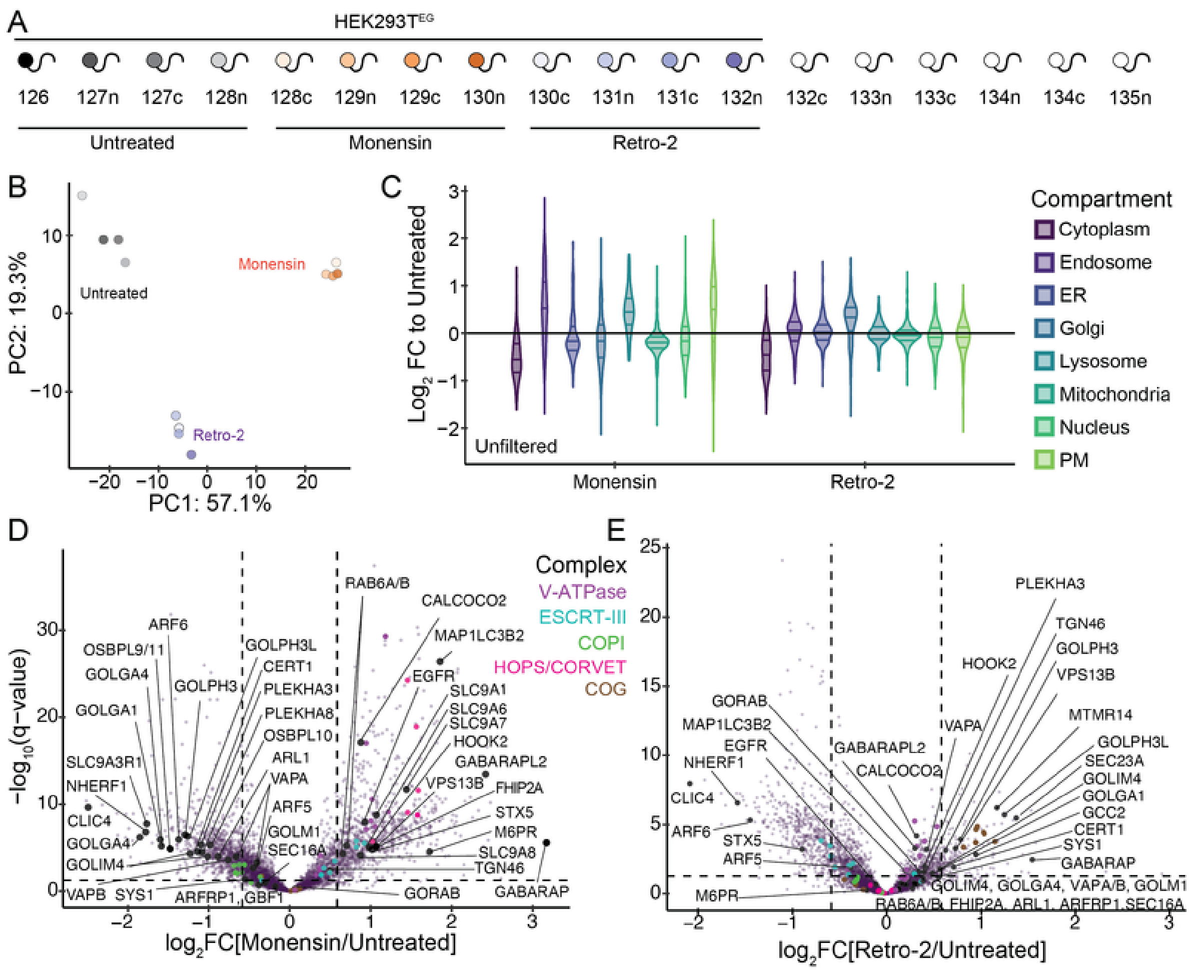

Figure 5—Supporting Figure 4.

(A) TMTplex experimental design. Cells were treated with the indicated compounds (monensin, 10 µM, 2 h; retro-2, 25 µM, 2 h) prior to GolgiIP on anti-HA magnetic beads. No background control was included in this experiment.

(B) Principal component analysis (PCA) colored as in (A) with all channels included. Replicates of a given condition correlate well.

(C) Violin plots depicting the GolgiIP $\log_2$ fold change (FC) values for the indicated subcellular compartment in aggregate for the indicated treatment compared to the untreated control (HEK293T^{EG}).

(D-E) GolgiIP volcano plots for (D) monensin (10 µM, 2 h)-treated or (E) retro-2 (25 µM, 2 h)-treated versus untreated HEK293T^{EG} cells. Protein complexes are colored as indicated. *P* values were calculated from the Student's t-test (two sided) and adjusted for multiple hypothesis correction using the Benjamini–Hochberg approach (q-value) with MSstats. Data represent n=4 biological replicates.

To Figure 5: https://harperlab.pubpub.org/pub/nlrp3#nkrmiksruzv

bioRxiv preprint doi: https://doi.org/10.1101/2024.04.19.590338; this version posted April 20, 2024. The copyright holder for this preprint (which was not certified by peer review) is the author/funder, who has granted bioRxiv a license to display the preprint in perpetuity. It is made available under aCC-BY 4.0 International license.

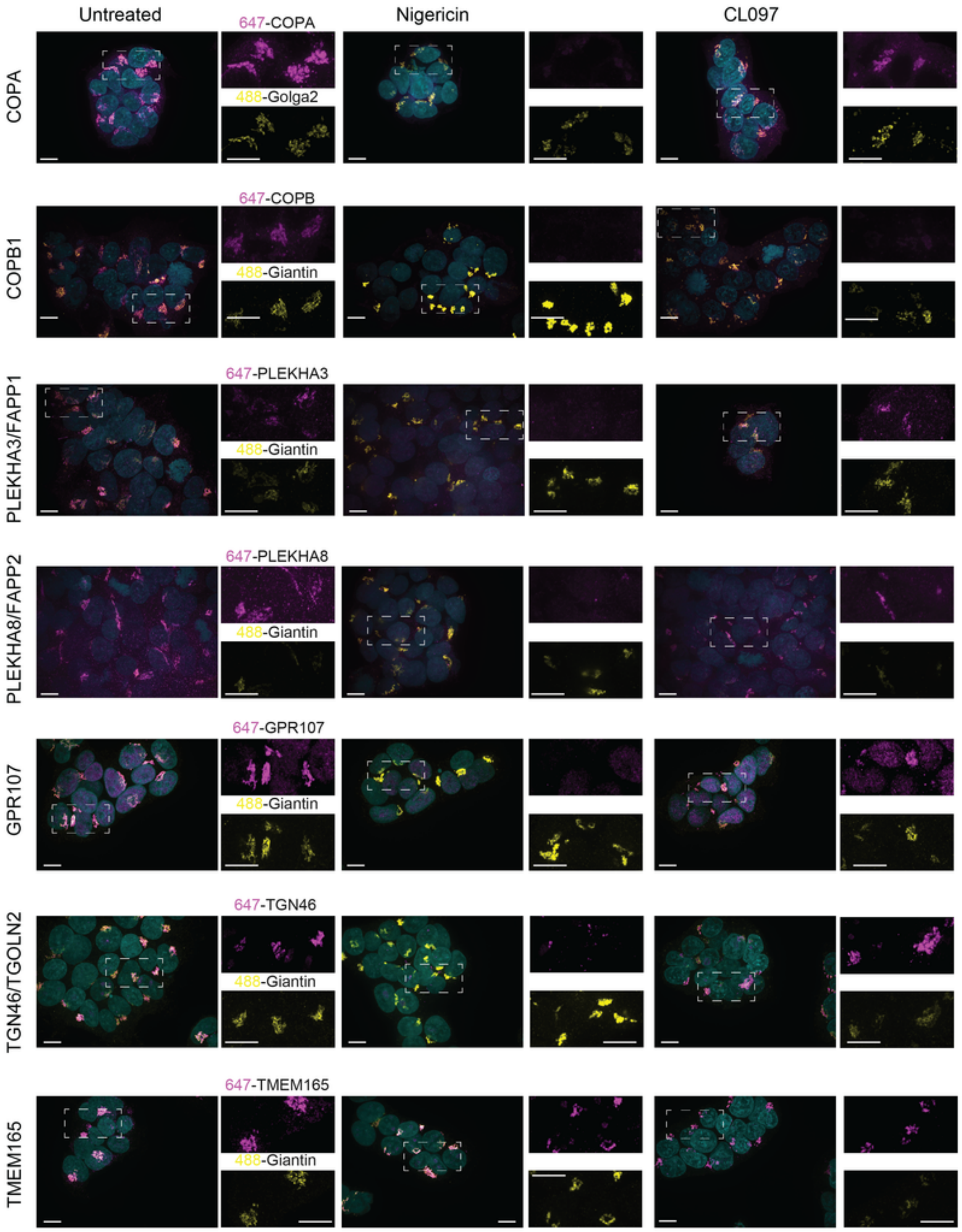

Figure 5—Supporting Figure 5. Golgi localization of COPA/B1, PLEKHA3/8, GPR107, TGN46, and TMEM165 in response to inflammasome agonists.
HEK293T cells were treated with nigericin (20 μM, 30 min) or CL097 (75 μg/mL, 1 h), fixed, and immunostained with the indicated antibodies (488 and 647 channels, colored as indicated) and Hoechst (405 channel, cyan). Giantin and Golga2 are well established Golgi markers. Maximum intensity projection images (z=8 μM, 29 steps), representative of n > 6 fields of view from at least two independent experiments. Scale bars, 10 μm.
To Figure 5: https://harperlab.pubpub.org/pub/nlrp3#nkrmiksruzv

Figure 6 supporting figures

bioRxiv preprint doi: https://doi.org/10.1101/2024.04.19.590338; this version posted April 20, 2024. The copyright holder for this preprint (which was not certified by peer review) is the author/funder, who has granted bioRxiv a license to display the preprint in perpetuity. It is made available under aCC-BY 4.0 International license.

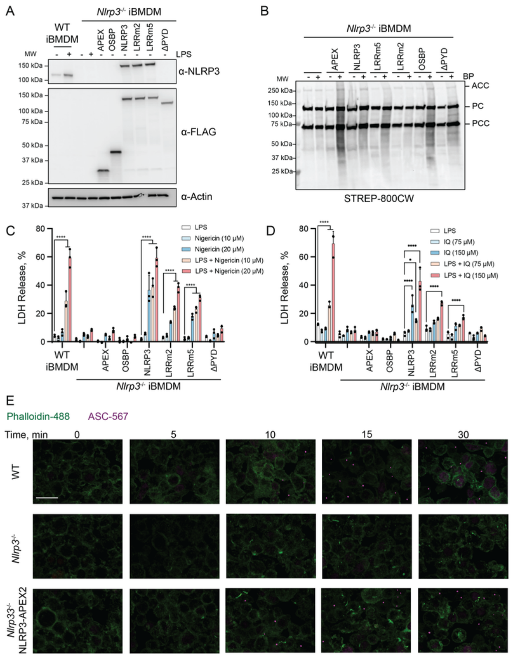

Figure 6—Supporting Figure 1. Validation of Nlrp3-APEX2 reconstitution.

(A) Validation of stable Nlrp3-APEX2 reconstitution at levels similar to LPS-primed WT iBMDMs, following two FACS sorts. Immunoblots with the indicated antibodies for WT and *Nlrp3*$^{-/-}$ iBMDMs ± LPS (1 μg/mL, 4 h), in addition to *Nlrp3*$^{-/-}$ iBMDMs stably reconstituted with the indicated APEX2-FLAG construct. The cage-disrupting Nlrp3 mutations LRRm2 (N1008R, R1009E, E1010R, R1013E) and LRRm5 (H781E, Q782R, F785A) were reported in Andreeva *et al.* [37]. Nlrp3ΔPYD is I125M-END (no polybasic linker region). PH, pleckstrin homology domain.

(B) Validation of APEX2 biotinylation activity. APEX2-expressing and control *Nlrp3*$^{-/-}$ iBMDMs ± biotin phenol (BP, 500 μM, 45 min) prior to labeling with H_2O_2 (10 μM, 1 min). Lysates were probed with STREP-800CW to detect biotinylated proteins. ACC, acetyl-CoA carboxylase. PC, pyruvate carboxylase. PCC, propionyl-CoA carboxylase.

(C-D) Reconstituted Nlrp3-APEX2 rescues inflammasome activity. % LDH release (relative to cell culture media and a maximum release control) of *Nlrp3*$^{-/-}$ iBMDMs reconstituted with various APEX2 constructs. Cells were treated without LPS or primed with LPS (1 μg/mL) 4 h prior to treatment with (C) nigericin (indicated concentrations, 1 h) or (D) imiquimod (indicated concentrations, 2 h). Bar graphs represent mean ± standard deviation, n=3 replicates. *p<0.05, **p<0.01, ***p<0.001, ****p<0.0001 using a two-way ANOVA with Tukey's post-test.

(E) Timecourse of inflammasome speck assembly. The indicated primed (4 h LPS, 1 μg/mL) iBMDMs were treated with 20 μM Nigericin for the indicated periods of time, fixed, immunostained (Hoechst, Phalloidin, anti-ASC), and imaged. Maximum intensity projections are shown (z=8 μM, 29 steps). Scale bar (top left image) = 25 μm. Representative regions of n > 3 fields of view.

To Figure 6: https://harperlab.pubpub.org/pub/nlrp3#nywwrxbdiu9

bioRxiv preprint doi: https://doi.org/10.1101/2024.04.19.590338; this version posted April 20, 2024. The copyright holder for this preprint (which was not certified by peer review) is the author/funder, who has granted bioRxiv a license to display the preprint in perpetuity. It is made available under aCC-BY 4.0 International license.

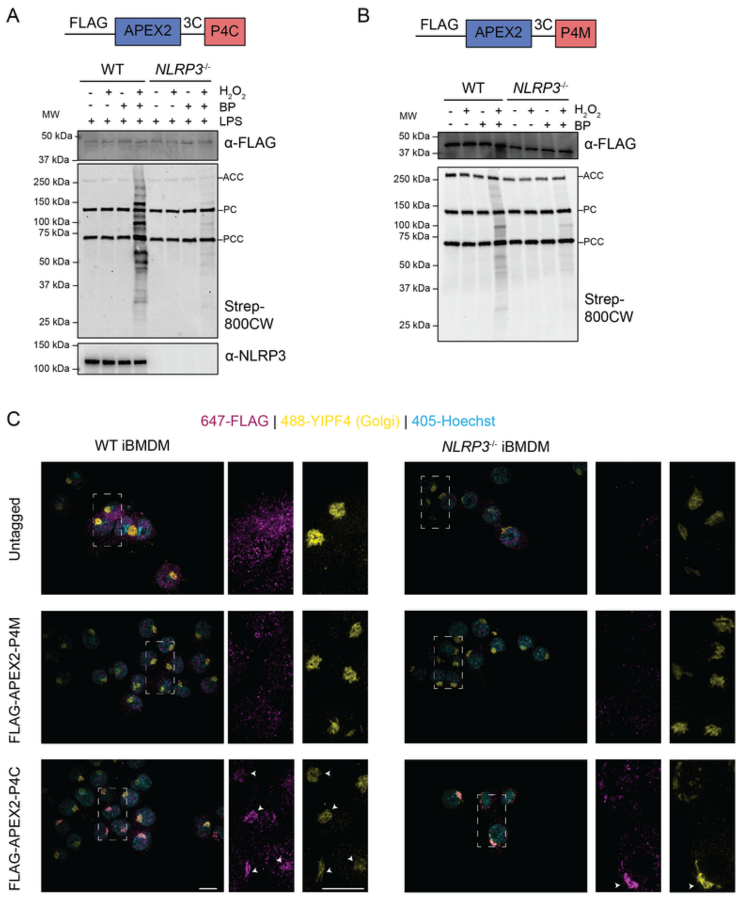

Figure 6—Supporting Figure 2. Validation of APEX2-P4C reconstitution. Validation of APEX2 biotinylation activity and PI4P biosensor Golgi localization.

(A) Immunoblots of LPS-primed (1 µg/mL, 4 h) WT and *Nlrp3*$^{-/-}$ iBMDMs reconstituted with FLAG-APEX2-P4C (expected 48 kDa). Cells were stimulated ± biotin phenol (BP, 500 µM, 30 min) followed by ± H_2O_2 (10 µM, 1 min) prior to quenching and harvesting cells. ACC, acetyl-CoA carboxylase. PC, pyruvate carboxylase. PCC, propionyl-CoA carboxylase. n=1 biological replicate.

(B) Immunoblots of WT and *Nlrp3*$^{-/-}$ iBMDMs reconstituted with FLAG-APEX2-P4M (expected 40 kDa). Cells were stimulated ± biotin phenol (BP, 500 µM, 30 min) followed by ± H_2O_2 (10 µM, 1 min) prior to quenching and harvesting cells. n=1 biological replicate.

(C) The P4C construct demonstrates Golgi localization by immunofluorescence of fixed cells whereas the P4M construct does not. The indicated primed (4 h LPS, 1 µg/mL) iBMDMs were fixed, immunostained as indicated, and imaged with confocal microscopy. Maximum intensity projection images (z=8 µM, 29 steps) are shown. Arrows indicate colocalized FLAG (P4C construct) and YIPF4 (Golgi marker) signal. Representative images from n > 6 fields of view. Scale bar (bottom left panels), 10 µm.

To Figure 6: https://harperlab.pubpub.org/pub/nlrp3#nywwrxbdiu9

bioRxiv preprint doi: https://doi.org/10.1101/2024.04.19.590338; this version posted April 20, 2024. The copyright holder for this preprint (which was not certified by peer review) is the author/funder, who has granted bioRxiv a license to display the preprint in perpetuity. It is made available under aCC-BY 4.0 International license.

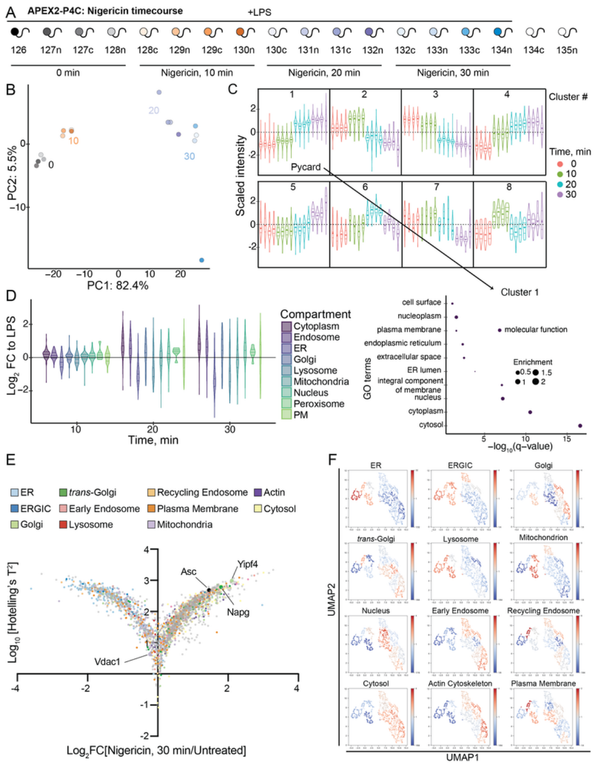

Figure 6—Supporting Figure 3. Supplemental data for the APEX2-P4C nigericin timecourse.
(A) APEX2-P4C nigericin timecourse experimental design. Cells were primed with LPS (1 μg/mL, 4 h) then treated with nigericin (20 μM; 0, 10, 20, 30 min) prior to labeling with H_2O_2 (10 μM, 1 min). All conditions were also treated with biotin phenol (500 μM, 45 min) prior to labeling.
(B) Principal component analysis (PCA) colored as in (A), showing timecourse-dependent replicate separation.
(C) Hierarchical clusters of ANOVA-significant proteins within the dataset. The behavior of each cluster is shown with violin plots of the aggregated data, replicates colored as indicated. The cluster containing Pycard (Asc) is annotated. GO-term analysis is shown for the Asc-containing cluster.
(D) Violin plots depicting the $\log_2$FC values for ER proteins detected in each experiment. Notably, proximity to ER proteins decreased rapidly.
(E) Multivariate empirical Bayes analysis for total APEX2 data over the 30-minute stimulation timecourse. Plot displays $\log_{10}$(time course Hotelling's T^2 statistic) versus $\log_2$FC(30 min nigericin versus unstimulated). Proteins are colored by organelle annotation as indicated.
(F) ANOVA-significant proteins the APEX2 timecourse were clustered with the Leiden algorithm. These clusters are colored as annotated on UMAP embeddings of the data by organelle enrichment. Organellar annotations for E,F reported in Hein*, Peng*, Todorova*, McCarthy*, Kim*, and Liu* *et al.* (graph-based annotations) [84].
To Figure 6: https://harperlab.pubpub.org/pub/nlrp3#nywwrxbdiu9

bioRxiv preprint doi: https://doi.org/10.1101/2024.04.19.590338; this version posted April 20, 2024. The copyright holder for this preprint (which was not certified by peer review) is the author/funder, who has granted bioRxiv a license to display the preprint in perpetuity. It is made available under aCC-BY 4.0 International license.

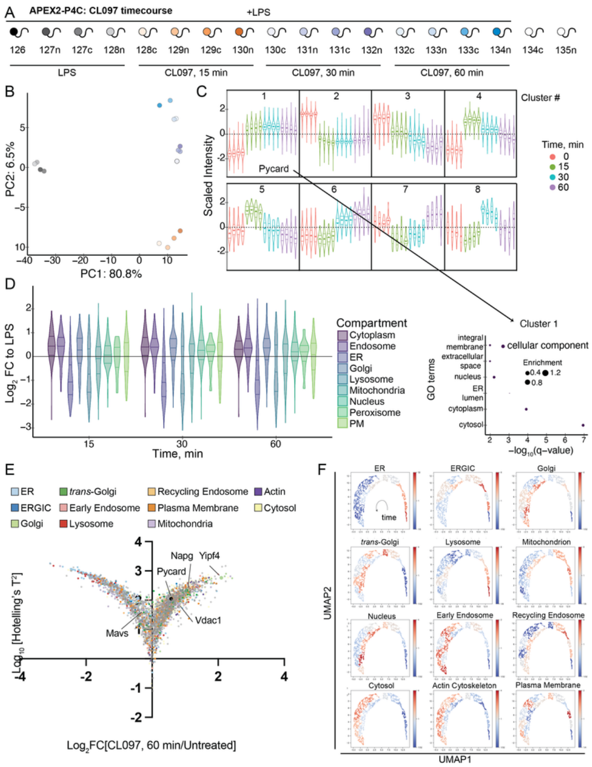

Figure 6—Supporting Figure 4. Supplemental data for the APEX2-P4C CL097 timecourse.
(A) APEX2-P4C CL097 timecourse experimental design. Cells were primed with LPS (1 μg/mL, 4 h) then treated with CL097 (75 μg/mL; 0, 15, 30, 60 min) prior to labeling with H_2O_2 (10 μM, 1 min). All conditions were also treated with biotin phenol (500 μM, 45 min) prior to labeling.
(B) Principal component analysis (PCA) colored as in (A), showing timecourse-dependent replicate separation.
(C) Hierarchical clusters of ANOVA-significant proteins within the dataset. The behavior of each cluster is shown with violin plots of the aggregated data, replicates colored as indicated. The cluster containing Pycard (Asc) is annotated. GO-term analysis is shown for the Asc-containing cluster.
(D) Violin plots depicting the $\log_2$FC values for ER proteins detected in each experiment. Notably, proximity to ER and lysosomal proteins decreased rapidly.
(E) Multivariate empirical Bayes analysis for total APEX2 data over the 60-minute stimulation timecourse. Plot displays $\log_{10}$(time course Hotelling's T^2 statistic) versus $\log_2$(60 min CL097 versus unstimulated). Proteins are colored by organelle annotation as indicated.
(F) ANOVA-significant proteins the APEX2 timecourse were clustered with the Leiden algorithm. These clusters are colored as annotated on UMAP embeddings of the data by organelle enrichment. Organellar annotations for E,F reported in Hein*, Peng*, Todorova*, McCarthy*, Kim*, and Liu* *et al*. (graph-based annotations) [84].
To Figure 6: https://harperlab.pubpub.org/pub/nlrp3#nywwrxbdiu9

bioRxiv preprint doi: https://doi.org/10.1101/2024.04.19.590338; this version posted April 20, 2024. The copyright holder for this preprint (which was not certified by peer review) is the author/funder, who has granted bioRxiv a license to display the preprint in perpetuity. It is made available under aCC-BY 4.0 International license.

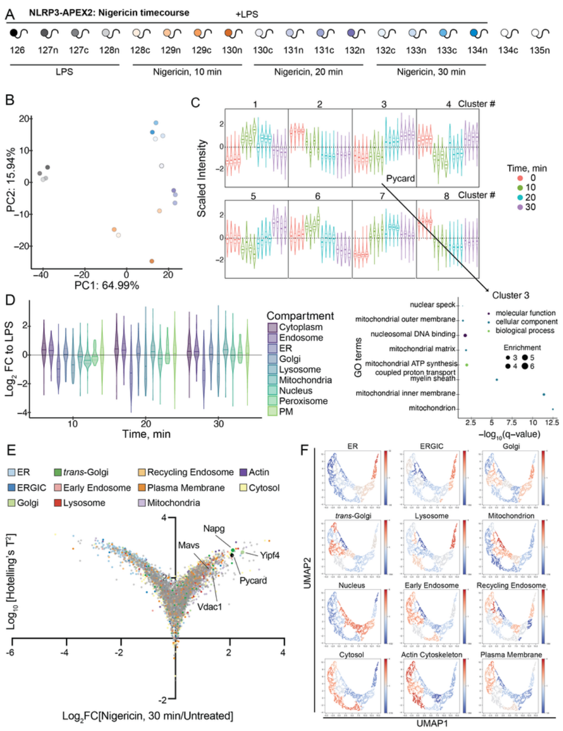

Figure 6—Supporting Figure 5. Supplemental data for the Nlrp3-APEX2 nigericin timecourse.

A) Nlrp3-APEX nigericin timecourse experimental design. Cells were primed with LPS (1 µg/mL, 4 h) then treated with nigericin (20 µM; 0, 10, 20, 30 min) prior to labeling with H_2O_2 (10 µM, 1 min). All conditions were also treated with biotin phenol (500 µM, 45 min) prior to labeling.

(B) Principal component analysis (PCA) colored as in (A), showing timecourse-dependent replicate separation.

(C) Hierarchical clusters of ANOVA-significant proteins within the dataset. The behavior of each cluster is shown with violin plots of the aggregated data, replicates colored as indicated. The cluster containing Pycard (Asc) is annotated. GO-term analysis is shown for the Asc-containing cluster.

(D) Violin plots depicting the $\log_2$FC values for ER proteins detected in each experiment. Notably, proximity to ER and lysosomal proteins decreased rapidly.

(E) Multivariate empirical Bayes analysis for total APEX2 data over the 30-minute stimulation timecourse. Plot displays $\log_{10}$(time course Hotelling's T^2 statistic) versus $\log_2$(30 min nigericin versus unstimulated). Proteins are colored by organelle annotation as indicated.

(F) ANOVA-significant proteins the APEX2 timecourse were clustered with the Leiden algorithm. These clusters are colored as annotated on UMAP embeddings of the data by organelle enrichment. Organellar annotations for E,F reported in Hein*, Peng*, Todorova*, McCarthy*, Kim*, and Liu* *et al*. (graph-based annotations) [84].

To Figure 6: https://harperlab.pubpub.org/pub/nlrp3#nywwrxbdiu9

bioRxiv preprint doi: https://doi.org/10.1101/2024.04.19.590338; this version posted April 20, 2024. The copyright holder for this preprint (which was not certified by peer review) is the author/funder, who has granted bioRxiv a license to display the preprint in perpetuity. It is made available under aCC-BY 4.0 International license.

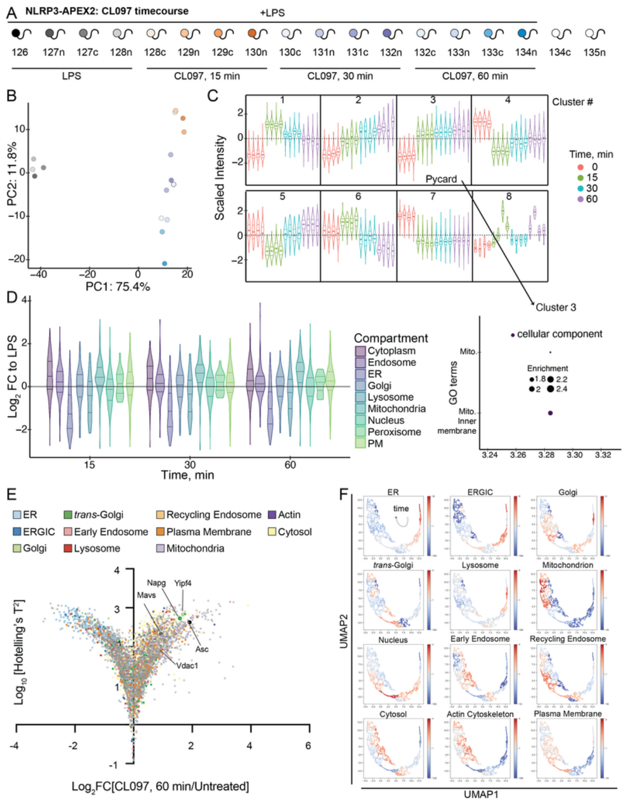

Figure 6—Supporting Figure 6. Supplemental data for the Nlrp3-APEX2 CL097 timecourse. (A) Nlrp3-APEX2 CL097 timecourse experimental design. Cells were primed with LPS (1 μg/mL, 4 h) then treated with CL097 (75 μg/mL; 0, 15, 30, 60 min) prior to labeling with H_2O_2 (10 μM, 1 min). All conditions were also treated with biotin phenol (500 μM, 45 min) prior to labeling.

(B) Principal component analysis (PCA) colored as in (A), showing timecourse-dependent replicate separation.

(C) Hierarchical clusters of ANOVA-significant proteins within the dataset. The behavior of each cluster is shown with violin plots of the aggregated data, replicates colored as indicated. The cluster containing Pycard (Asc) is annotated. GO-term analysis is shown for the Asc-containing cluster.

(D) Violin plots depicting the log_2FC values for ER proteins detected in each experiment. Notably, proximity to ER and lysosomal proteins decrease rapidly.

(E) Multivariate empirical Bayes analysis for total APEX2 data over the 60-minute stimulation timecourse. Plot displays log_{10}(time course Hotelling's T^2 statistic) versus log_2 (60 min CL097 versus unstimulated). Proteins are colored by organelle annotation as indicated.

(F) ANOVA-significant proteins the APEX2 timecourse were clustered with the Leiden algorithm. These clusters are colored as annotated on UMAP embeddings of the data by organelle enrichment. Organellar annotations for E,F reported in Hein*, Peng*, Todorova*, McCarthy*, Kim*, and Liu* *et al*. (graph-based annotations) [84].

bioRxiv preprint doi: https://doi.org/10.1101/2024.04.19.590338; this version posted April 20, 2024. The copyright holder for this preprint (which was not certified by peer review) is the author/funder, who has granted bioRxiv a license to display the preprint in perpetuity. It is made available under aCC-BY 4.0 International license.

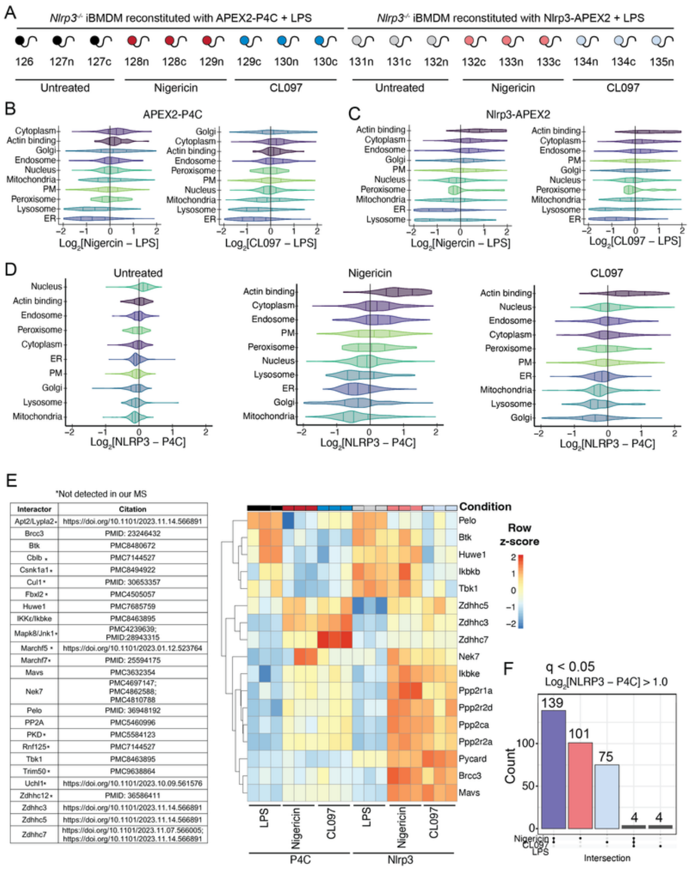

Figure 7—Supporting Figure 1. Supplemental data for proximity labeling with P4C and Nlrp3 in the same TMTplex.

(A) Combined APEX2 experiment design. *Nlrp3*$^{-/-}$ iBMDMs expressing either APEX2-P4C (background control) or Nlrp3-APEX2 were primed with LPS (1 µg/mL, 4 h) then treated with nigericin (20 µM, 20 min) or CL097 (75 µg/mL, 25 min) prior to labeling with H_2O_2 (10 µM, 1 min). All conditions were also treated with biotin phenol (500 µM, 45 min) prior to labeling.
(B-C) Violin plots depicting the log_2FC values across the indicated subcellular compartments for the given treatment condition versus untreated cells. Comparisons are either for *Nlrp3*$^{-/-}$ iBMDMs expressing (B) APEX2-P4C or (C) Nlrp3-APEX2.
(D) Violin plots depicting the log_2FC values across the indicated subcellular compartments for the indicated Nlrp3-APEX2 condition to its matched P4C-APEX2 background condition.
(E) Summary of high confidence Nlrp3 interactors from the literature (left) and a heatmap of those interactors present in the dataset (right), showing row z-score as annotated.
(F) UpSet plot of proteins enriched in Nlrp3-APEX2 over APEX2-P4C (any matched comparison of Nlrp3/P4C: $q < 0.05$, $Log_2FC > 1.0$). Intersections show multiple instances of Nlrp3-APEX2 enrichment over the APEX2-P4C background.
To Figure 7: https://harperlab.pubpub.org/pub/nlrp3#njz6bdmzuz6
